# Calcium-Sensing Receptors of Human Neural Cells Play Crucial Roles in Alzheimer's Disease

**DOI:** 10.3389/fphys.2016.00134

**Published:** 2016-04-26

**Authors:** Anna Chiarini, Ubaldo Armato, Daisong Liu, Ilaria Dal Prà

**Affiliations:** ^1^Human Histology and Embryology Unit, University of Verona Medical SchoolVerona, Italy; ^2^Proteomics Laboratory, Institute for Burn Research, Third Military Medical UniversityChongqing, China

**Keywords:** calcium-sensing receptor, calcilytic, human, neurons, astrocytes, oligodendrocytes, microglia, Alzheimer's disease

## Abstract

In aged subjects, late-onset Alzheimer's disease (LOAD) starts in the lateral entorhinal *allocortex* where a failure of clearance mechanisms triggers an accumulation of neurotoxic amyloid-β_42_ oligomers (Aβ_42_-os). In neurons and astrocytes, Aβ_42_-os enhance the transcription of Aβ precursor protein (APP) and β-secretase/BACE1 genes. Thus, by acting together with γ-secretase, the surpluses of APP and BACE1 amplify the endogenous production of Aβ_42_-os which pile up, damage mitochondria, and are oversecreted. At the plasmalemma, exogenous Aβ_42_-os bind neurons' and astrocytes' calcium-sensing receptors (CaSRs) activating a set of intracellular signaling pathways which upkeep Aβ_42_-os intracellular accumulation and oversecretion by hindering Aβ_42_-os proteolysis. In addition, Aβ_42_-os accumulating in the extracellular milieu spread and reach mounting numbers of adjacent and remoter teams of neurons and astrocytes which in turn are recruited, again via Aβ_42_-os•CaSR-governed mechanisms, to produce and release additional Aβ_42_-os amounts. This relentless self-sustaining mechanism drives AD progression toward upper cortical areas. Later on accumulating Aβ_42_-os elicit the advent of hyperphosphorylated (p)-Tau oligomers which acting together with Aβ_42_-os and other glial neurotoxins cooperatively destroy wider and wider cognition-related cortical areas. In parallel, Aβ_42_-os•CaSR signals also elicit an excess production and secretion of nitric oxide and vascular endothelial growth factor-A from astrocytes, of Aβ_42_-os and myelin basic protein from oligodendrocytes, and of proinflammatory cytokines, nitric oxide and (likely) Aβ_42_-os from microglia. Activated astrocytes and microglia survive the toxic onslaught, whereas neurons and oligodendrocytes increasingly die. However, we have shown that highly selective allosteric CaSR antagonists (calcilytics), like NPS 2143 and NPS 89626, efficiently suppress all the neurotoxic effects Aβ_42_-os•CaSR signaling drives in cultured cortical untransformed human neurons and astrocytes. In fact, calcilytics increase Aβ_42_ proteolysis and discontinue the oversecretion of Aβ_42_-os, nitric oxide, and vascular endothelial growth factor-A from both astrocytes and neurons. Seemingly, calcilytics would also benefit the other types of glial cells and cerebrovascular cells otherwise damaged by the effects of Aβ_42_-os•CaSR signaling. Thus, given at amnestic minor cognitive impairment (aMCI) or initial symptomatic stages, calcilytics could prevent or terminate the propagation of LOAD neuropathology and preserve human neurons' viability and hence patients' cognitive abilities.

## Alzheimer's disease (AD): an introduction

During the last decades, human lifespan has lengthened due to progress in medical knowledge and improvements in nutrition and hygiene. Unfortunately, this has been paralleled with an increased prevalence of age-related ailments, including neurodegenerative diseases, which have adversely impacted the quality of life. The *sporadic* or *late-onset Alzheimer's disease* (*LOAD*) is the most prevalent of these dementias striking ~60 million people worldwide, half of which in the European Union and United States (Alzheimer's Association, 2012). AD has a lengthy (20–45 years) asymptomatic or preclinical phase, followed by an amnestic minor cognitive impairment phase (aMCI: 2–6 years) that in most subjects evolves into the terminal fully symptomatic phase (6–12 years; Selkoe, 2008a,b; Sperling et al., 2011). AD's less frequent (1–5% of all cases) *early onset* (around 60 years) *familial* (autosomal dominant) form (*EOFAD*) is caused by mutations in genes encoding the amyloid precursor protein (APP) or presenilin 1 (PSEN1) or presenilin 2 (PSEN2). These mutations trigger excess production and secretion of amyloid-β peptides (Aβs) and formation of toxic oligomers (Aβ-os) and polymers (fibrils). Most EOFAD cases result from PSEN1 mutations, those from APP and PSEN2 mutations being rarer (Selkoe, 2008a,b). The dramatic effects elicited by the Aβs excess due to such mutations have inspired the “*amyloid cascade hypothesis*” of AD which posits that Aβ-os precede the manifestation of toxic hyperphosphorylated (p)-Tau proteins and neurofibrillary tangles (NFTs) (Hardy and Selkoe, 2002; Selkoe, 2008a,b). Conversely, the “*Tau first hypothesis*” of AD posits that just the opposite happens (Attems et al., 2012; Braak and Del Tredici, 2013; Braak et al., 2013). An extended post-mortem survey revealed that AD cognitive decline is linked to both Aβs and p-Taues build-ups (Murray et al., 2015). However, Choi et al. (2014) provided evidence that, in a 3D human neural stem cells (NSCs) culture system, the accumulation of Aβ-os precedes any p-Tau/NFTs materialization thereby validating the “*amyloid cascade hypothesis*.” Bilousova et al. (2016) confirmed that this Aβ-os ⇒ p-Tau sequence occurs also in advanced AD stages, strengthening the view that an anti-amyloid therapy must be started in advance of the tauopathy onset.

**Figure 1:**
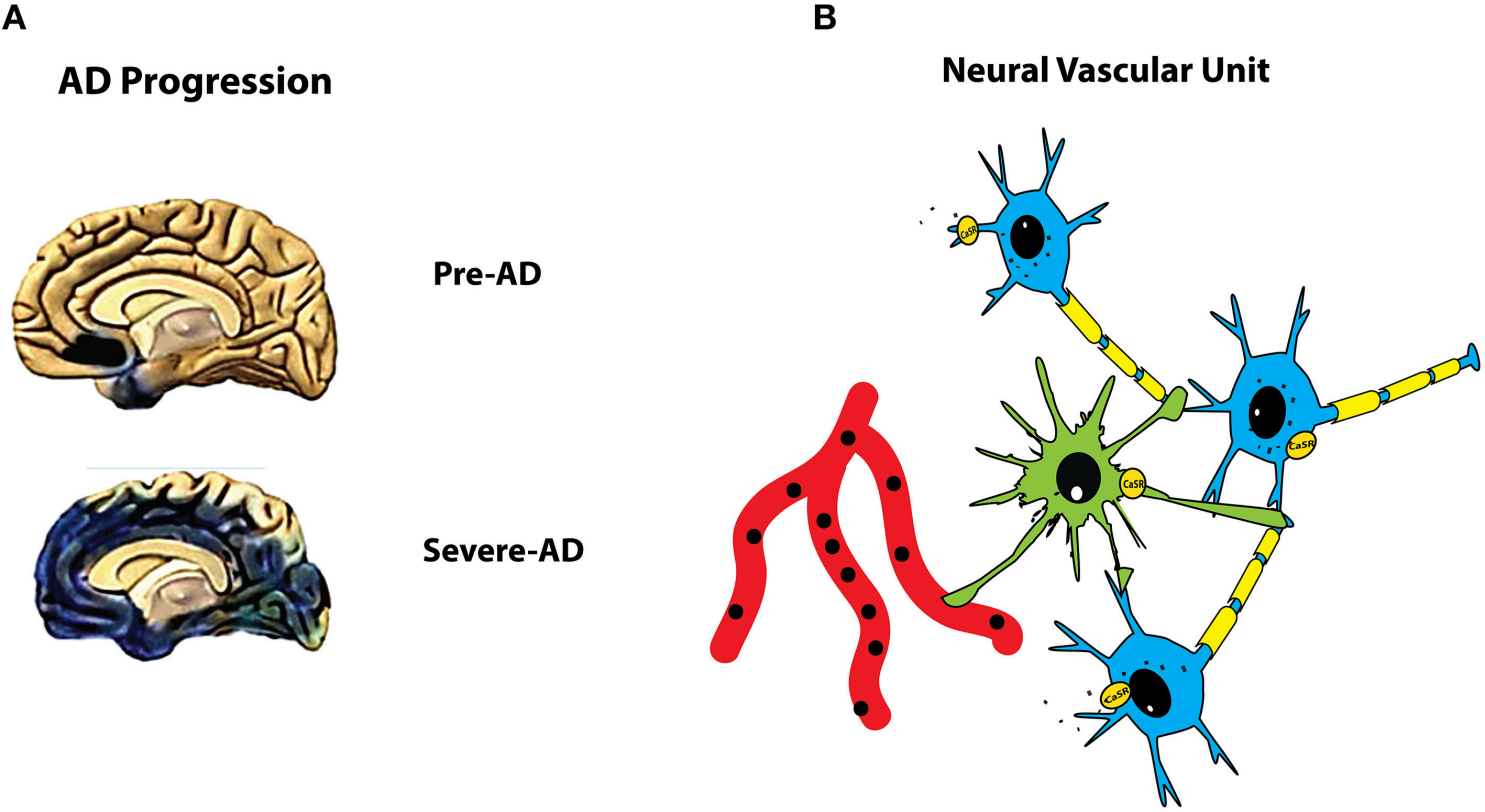
**(A)** Late-onset AD (LOAD) neuropathology affects increasingly wider cerebral cortical areas. LOAD is a spreading disease which starts from the layer II neurons of the lateral entorhinal *allocortex* (LEC) of the temporal lobe and expands progressively to cognition-related upper neocortical areas. Involved brain tissues undergo deep changes due to concurrent neurotoxic, inflammatory, oxidative, and hypoxic-ischemic processes driven by accumulating Aβ_42_-os and Aβ fibrils and later by p-Tau-os and causing the death of susceptible neurons. The diagram represents a view of the LOAD-affected areas (*in dark blue color*) from the medial-inferior hemispheric face at an early (*Pre-AD*), presymptomatic AD) and a late fully symptomatic stage of the illness. **(B)** The basic organization of the brain's neurovascular unit (NVU). NVUs are made up by cerebral astrocyte-neurons teams (ANTs) placed in close contact with capillary vessels. In this cartoon, a “master” astrocyte (*in green color*) emits numerous cytoplasmic processes (of which only a few are depicted here), the end-feet of which enshroud two neuronal synapses, touch the dendrite of a “client” neuron (*in blue color*), and cover a portion of the outer surface of a capillary vessel (*in red color*). The neuronal axons are endowed with myelin sheaths (*in yellow color*). Both neurons and the astrocyte express the CaSR (*yellow ovals*). By its placement, the astrocyte acts as a bridge between the capillary vessel and the neurons, provides the latter with nutrients brought up by the former, protects synapses, and partakes in the upkeep of the brain-blood barrier (BBB; not shown) functional integrity.

Albeit clinically both EOFAD and LOAD present with a similarly increasing memory failure, at variance with EOFAD's known mutations, LOAD's etiologic factors are manifold and controversial. The slow concurrence of several age-related metabolic and vascular defects presumably triggers LOAD by hindering the mechanisms which effect the brain's physiological clearance of Aβs (Domert et al., 2014). Two genetic factors only are known to aid LOAD's onset and progression, i.e., the heterozygous or homozygous presence of apolipoprotein E (APOE) ε4 allele(s) and TREM-2 mutations, especially the R47H one (Ising et al., 2015). AD's neuropathological hallmarks are accumulations of Aβs as senile plaques in the neuropil, intra-neuronal build-ups of p-Taues as insoluble NFTs, a chronic diffuse neuroinflammation, and the progressive death of neurons and oligodendrocytes. Such characteristics are detectable and more intense in wide cortical and subcortical regions starting at least 15 years ahead of EOFAD's clinical onset (Braak and Braak, 1991a; Armstrong, 2011; Benzinger et al., 2013). Conversely, LOAD starts from neuronal *foci* in the layer II of the lateral entorhinal cortex (LEC) of the middle temporal lobe in humans and AD-model transgenic (Tg) mice (Khan et al., 2014). Synaptically disconnected and deceasing neurons stuffed up with Aβs and NFTs appear first in the LEC *allocortex* and *subiculum*/CA1 areas (Braak and Braak, 1991a; Gómez-Isla et al., 1996; Khan et al., 2014) and later spread slowly to the parietal lobes and other cognition-related cortical areas of human AD brains (reviewed in Dal Prà et al., 2015a; Figure 1A). Remarkably, LEC is the portal through which the perforant pathway conveys multimodal data illustrating events of the outside world to the memory-recording hippocampus (Klemm, 2014). Next, the hippocampal *allocortex* and prefrontal *neocortex* mutually interact through the LEC to consolidate integrated memories (Klemm, 2014). These bidirectional exchanges are compromised by the ravages LEC suffers at the aMCI stage of LOAD. Hence, the first clinical harbingers of LOAD are worsening failures of the declarative memory.

The present lack of an anti-AD beneficial therapy is due to several concurrent causes: (i) LOAD's etiology is still hotly debated; (ii) EOFAD and LOAD are diseases typical of the human central nervous system (CNS) whose features can be only partially modeled in animals because of the huge differences in brain structures and cellular functions. The significant losses of hippocampal and neocortical neurons while the astrocytes survive are emblematic of human AD. Conversely, in most Tg rodents AD-models neurons are spared whereas astrocytes undergo an earlier cytotoxic injury and death. That's why any drug reportedly “successful” in AD-model animals has failed the test of clinical trials (reviewed in Han et al., 2015); and (iii) most previous clinical trials of candidate anti-AD drugs recruited patients already at the symptomatic stage of EOFAD or LOAD, *viz*. their cognitive cortical areas had already undergone irretrievable damage (Cummings et al., 2014). These failures have taught at a high cost that any anti-LOAD therapy must be started as early as possible, i.e., at the aMCI stage or just a little later for the time being (or earlier when it will be feasible). Hitherto, no specific marker of the preclinical stage of LOAD has been validated. However, changes in cerebrospinal fluid total Tau protein, p-Tau, and Aβ_42_ levels, novel techniques of high-resolution functional Magnetic Resonance Imaging (fMRI), and genetic risk profiling show the potential of a future early diagnosis (Nordberg, 2015).

## Aβs and AD neuropathology

In healthy human brains, neurons steadily produce physiologically low amounts (~200 pM) of harmless monomeric Aβ_42_s and release them during synaptic activity (Puzzo et al., 2008, 2011; Abramov et al., 2009; Garcia-Osta and Alberini, 2009). Aβs are synthesized via sequential enzymatic cleaving of the transmembrane Aβ precursor protein (APP) by BACE1/β-secretase (β-S) and γ-secretase (γ-S) (Takami and Funamoto, 2012). According to the “classical” view, only neurons express β-S in normal brains, whereas astrocytes do it only when hit by stressful insults (Kimura et al., 2005; Lee et al., 2014). However, proliferatively quiescent untreated normofunctioning adult human astrocytes (NAHAs) isolated from surgical leftovers of the temporal lobe cortex and cultured *in vitro* exhibit, at variance with rodent astrocytes, low basal levels of β-S and γ-S activity and hence produce and release trivial amounts of Aβ_42_ and Aβ_40_ (Dal Prà et al., 2011; Armato et al., 2013a).

Normally, the production of Aβ_40_ prevails (90%) on that of Aβ_42_ (10%), but in AD the Aβ_42_/Aβ_40_ ratio shifts in favor of Aβ_42_ (Masters and Selkoe, 2012). Aβ_42_'s two C-terminal hydrophobic amino acids, Ala and Ile, cause its greater proclivity to form aggregates and resist proteolysis with respect to Aβ_40_ (Kim and Hect, 2005; Masters and Selkoe, 2012). At safe pM concentrations Aβ_42_ monomers play important trophic functions by: (a) inducing an enhanced expression of proteins related to insulin-like growth factor (IGF) function or transcription factor (TF) regulation (IGFBP3/5, and Lim only domain protein 4, respectively); (b) favoring adult neurogenesis in the subgranular zone of dentate gyrus; (c) modulating synaptic plasticity, long-term potentiation (LTP), and memories recording in the hippocampus; (d) sealing blood vessels to preserve blood-brain barrier (BBB) integrity; and (e) fine-tuning Ca^2+^ homeostasis by binding α7-nicotinic acetylcholine receptors (α7-nAChRs) and enhancing *intra*cellular Ca^2+^ signals without triggering *inter*cellular Ca^2+^ waves in astrocytes. Thus, Aβ_42_ monomers assist in the mutual modulation of neuron-astrocyte signals promoting long-term potentiation (LTP) and memory storing (Plant et al., 2003; Koudinov and Berezov, 2004; Puzzo et al., 2008, 2011; Garcia-Osta and Alberini, 2009; Morley et al., 2010; Cárdenas-Aguayo et al., 2014; Lee et al., 2014; Storck et al., 2016). Aβ_42_s are kept at physiological pM levels via the activity of proteases like insulin-degrading enzyme, neprilysin, angiotensin-converting enzyme, endothelin-converting enzyme, and the ubiquitin-proteasome system (López Salon et al., 2003; Wang et al., 2006). Additional Aβ-disposing mechanisms are microglial phagocytosis, and dumping into the circulating blood through the α2-macroglobulin receptor/low density lipoprotein receptor-related protein 1 (LRP1) (Storck et al., 2016).

Such monomeric Aβ-clearing mechanisms become inadequate when mutations of *APP* or *PSEN1* or *PSEN2* genes cause an overproduction of Aβs as in EOFAD or when they significantly decline with age and fail in LOAD (Tarasoff-Conway et al., 2015). The resulting accumulation of Aβ_42_s triggers the assembling of Aβ_42_ monomers into an assortment of toxic Aβ_42_ oligomers (Aβ_42_-os) of growing sizes eventually forming Aβ fibrils (Braak and Braak, 1991b; Mawuenyega et al., 2010; Masters and Selkoe, 2012; Lesnè et al., 2013). In addition, the generation of long fatty acid–derived oligomers (LFA-os) via a prion-like mechanism (Kumar and Walter, 2011; Kumar A. et al., 2012), the increasing presence of the Aβ_43_ isoform (Sandebring et al., 2013), and the Aβ-phosphorylating activity of membrane-bound or extracellular protein kinase A (Kumar and Walter, 2011; Kumar S. et al., 2012) accelerate the rate of Aβ-os assembly, reduce their proteolytic or microglia-mediated clearance, and step up their neurotoxicity. Another toxic species is pyroglutamate (pE)-Aβ_3−42_, which amounts to ~20% of the total Aβs in AD brains, but is missing among the Aβs extracted from aged yet cognitively normal brains (Gunn et al., 2010; Jawhar et al., 2011). In AD-developing human brains, pE-Aβ_3−42_ engenders pure or mixed (with other Aβs) highly toxic oligomers, the amount of which tightly correlates with the actual rate of cognitive decline (Morawski et al., 2014). Additionally, the N-truncated Aβ_4−42_ also abounds in AD brains and spawns stable Aβ_4−42_-os which are as neurotoxic as Aβ_1−42_-os and pE-Aβ_3−42_-os *in vitro* and in the mouse Tg4-42 transgenic line (Bouter et al., 2013). Moreover, interactions with cell membranes increase the aggregation rate of Aβ_42_-os and produce amyloid pores and Ca^2+^-permeable channels resulting in an intracellular Ca^2+^ dyshomeostasis promoting the neurodegeneration (Mattson, 2007; Kawahara, 2010; Zhao et al., 2012; Berridge, 2014). However, being pathologically bound and activated by Aβ_42_-os, the calcium-sensing receptor (CaSR) expressed by all types of neural cells is also involved in AD development via mechanisms implicating much more than Ca^2+^ influxes.

## Interactions between neurons and astrocytes in LOAD

Neurons and astrocytes derive from embryonic radial glia acting as neural stem cells (NSCs) during development (Bonfanti and Peretto, 2007). Accumulating evidence has shown that human cortical astrocytes remarkably differ from their rodent counterparts. They are bulkier, own 10-fold more numerous primary processes, include the entirely new cortical polar and interlaminar subtypes, exhibit a different transcriptome as assessed by genome-wide unbiased comparisons, govern much broader synaptic domains, and perform more intense and complex metabolic tasks, e.g., faster Ca^2+^ waves propagation, than rodents' counterparts (Oberheim et al., 2006, 2009, 2012; Sherwood et al., 2006; Tsai et al., 2012; Zhang et al., 2016). Astrocytes' evolutionary changes have affected both human brain physiology and neuropathology, including AD and other neurodegenerative disorders. The increased learning capacity and activity-dependent plasticity of mouse brains engrafted with human astrocytes confirms this view (Han et al., 2013). Human brain evolutive changes prevent AD-model animals from fully emulating human LOAD. This hampers any successful translation of pharmacological results reaped from AD-model animals to human clinical settings (Cummings et al., 2014; Han et al., 2015). Astrocytes' roles in AD progression deserve a careful consideration. Such cells are more numerous (from 1.7- to 2.2-fold at least) than neurons, form gap junction-connected networks, partake in the assembly of tripartite synapses, and tightly nestle and chemically insulate neurons with which physiologically trade several indispensable compounds (Ullian et al., 2001). Each “*master*” astrocyte functionally integrates with up to a 30-odd “*client*” neurons forming *astrocyte-neuron teams* (*ANTs*; reviewed in Araque and Navarrete, 2010; Giaume et al., 2010; Halassa and Haydon, 2010). Neighboring ANTs are reciprocally connected via gap junctions astrocytes' processes bear. Other astrocytes processes get in touch by means of their end-feet with the walls of cerebral micro vessels forming physiologically integrated *neurovascular units* (*NVUs*) (Figure 1B; reviewed in Dal Prà et al., 2014b; Nelson et al., 2016). Physiologically, the synapses of ANTs “*client*” neurons are induced and stabilized by the shrouding end-feet of their “*master*” astrocytes. Moreover, the synapses pertaining to a single neuron can also be enveloped by the processes end-feet of astrocytes pertaining to neighboring ANTs. Importantly, the astrocytes of connected ANTs promote or reduce the release of neurotransmitters into the synapses they wrap thus modulating neural transmission by (a) sweeping up spilled over glutamate and K^+^; (b) releasing “*gliotransmitters*” like glutamate, ATP, D-serine, γ-amino butyric acid (GABA), and taurine; and (c) letting out or taking up, respectively, Ca^2+^ ions during their Ca^2+^ waves (Antanitus, 1998; Bushong et al., 2002; Kettenmann and Ransom, 2013; Gundersen et al., 2015). The term *infotropism* defines the control of neurotransmitter release and hence of synaptic function by the astrocytes (Antanitus, 1998). Astrocytes' activation is coupled with *intra*cellular Ca^2+^ transients and *inter*cellular gap-junction-mediated Ca^2+^ waves and triggers both locally and remotely the secretion of gliotransmitters modulating astrocyte-astrocyte and astrocyte-neuron signaling (Lee et al., 2014). Moreover, astrocytes express receptors for other neurotransmitters—like purines, GABA, and N-methyl-D-aspartate (NMDA)—and control extracellular ion levels (e.g., K^+^), pH, and water volume (reviewed in Kettenmann and Ransom, 2013). Because of these distinctive properties, human astrocytes likely play a role as neuronal partners in learning, memory, and cognition—all functions progressively lost in AD.

In AD, extracellularly accumulating Aβ-os and Aβ fibrils contact all cellular members of ANTs and NVUs. In Tg AD-model rodents, while acting as wardens, astrocytes sweep extracellular Aβs by engulfing them via several Aβ-binding receptors, like LRP1 and LRP2/Megalin, and next proteolyse them. Eventually, ingested Aβs are toxic for the astrocytes which before dying discharge them back into the extracellular milieu. This promotes the assembly of smaller senile plaques which are rich in glial fibrillary acidic protein (GFAP; Wyss-Coray et al., 2003; Nagele et al., 2004; Pihlaja et al., 2008). Thus, an initially beneficial clearing of Aβs surpluses by the astrocytes eventually competes with and wrecks their role as supporters of neurons metabolism (Pihlaja et al., 2008; Araque and Navarrete, 2010; Giaume et al., 2010; Halassa and Haydon, 2010; Mulder et al., 2012). In 3 × Tg AD-model mice, as AD slowly yet inexorably progresses, the pattern of astrocytes' reactions changes. Astrocytes' processes rapidly wither and detach their shrouding end-feet from the tripartite synapses within the CA1 area and dentate gyrus. An early diffuse astrogliosis develops surrounding the senile plaques (Rodriguez-Vieitez et al., 2015).

Conversely, in human AD brains, astrocytes become hypertrophic, conserve their spatial domains, pierce with their processes Aβ senile plaques, lose part of their glutamate-metabolizing enzymes, over express GFAP, hyper polymerize actin, and make and release surplus amounts of cytokines and chemokines, such as S100β, TNF-α, IL-1β, IL-6, and IFN-γ-inducible protein-10 (IP-10). The sercreted chemokines induce circulating leukocytes to cross the BBB and sustain a chronic neuroinflammation (Perez et al., 2010). In aMCI patients, but not in healthy individuals, an astrogliosis can be detected (using the [11] CD-deprenyl marker and Positron Emission Tomography) which abates during the progression toward full-blown AD (Choo et al., 2014). This human AD-related astrogliosis co-occurs with oxidative stress, extracellular accumulation of glutamate and/or K^+^, dyslipidemia, and/or folate deficit (Rojo et al., 2008; Li et al., 2011).

A belief has been prevailing for a long time, i.e., only a transneuronal diffusion of neurotoxic Aβ-os happens in AD. This view was experimentally modeled in retinoic acid–differentiated human SH-SY5Y neurons (Nath et al., 2012; Hallbeck et al., 2013). Conversely, astrocytes' own production and secretion of Aβs as well as their potential contribution to AD progression was generally neglected. According to such a “classical” view, astrocytes only played the role of onlookers or at most of concierges cleansing neuronal debris and/or Aβ fibrils. Reports of astrocytes stuffed with Aβ_42_s in human brains with advanced LOAD strengthened this view (Nagele et al., 2004; Maragakis and Rothstein, 2006; Avila-Muñoz and Arias, 2014). Yet, because of their high numbers, even a token increase in astrocytes' Aβs secretion rate would remarkably raise brain's load of Aβs (Busciglio et al., 1993; Corbett and Buss, 2014). Nevertheless, recent studies have provided evidence that the intracerebral diffusion of Aβ_42_-os results from chemical interactions of astrocytes with neurons, oligodendrocytes, and microglia (Skaper et al., 2009; Bero et al., 2011; Dal Prà et al., 2015a). Such reciprocal exchanges *activate* the astrocytes which then express surpluses of APP and of β-S which act with γ-S to trigger an overproduction of Aβs in several Tg AD-model mice (Rossner et al., 2005). The effects of exogenous Aβs on mouse, rat, and human astrocytes and neurons have been studied *in vivo* and *in vitro*. Physiological patterns of astrocytes' intercellular Ca^2+^ waves and synchronous hyperactivity are changed in Tg AD-model animals (Kuchibhotla et al., 2009). When exposed to Aβ-os, newborn rat hippocampal astrocytes exhibited an increased intracellular Ca^2+^ concentration $\left({\left[{\text{Ca}}^{2+}\right]}_{\text{i}}\right)$. Hence, a Ca^2+^ dyshomeostasis occurs in the astrocytes activated by AD (reviewed in Abramov et al., 2003; Mattson and Chan, 2003; Bezprozvanny and Mattson, 2008; Berridge, 2014). Moreover, mouse astrocytes exposed to Aβ_25−35_ produced and secreted ceramide-stuffed exosomes (“*apoxosomes*”) and prostate apoptosis response-4 (PAR-4) protein which would trigger the apoptotic demise of nearby neurons releasing Aβs surpluses (Wang et al., 2012). In addition, primary cortical astrocytes from neonatal mouse pups treated with TNF-α + IFN-γ or Aβ_42_ (either in soluble or fibrillar form) raised the cells' levels of APP and β-S and their secretion rate of endogenous Aβ_40_ (yet, Aβ_42_ secretion was not assessed). The authors surmised that neuroinflammation triggers a feed-forward mechanism pushing the production of endogenous Aβs in mouse astrocytes (Zhao et al., 2011).

As mentioned above, β–S and γ–S exhibit a discrete basal activity in untreated (control) NAHAs (Armato et al., 2013a). Once exposed to exogenous fibrillary or soluble Aβ_25−35_—an Aβ_42_ proxy having the physical and biological features of Aβ_42_ (Kaminsky et al., 2010)—NAHAs start producing, accumulating, and secreting surplus Aβ_42_/Aβ_42_-os just as human cortical HCN-1A neurons do (Dal Prà et al., 2011; Armato et al., 2013a).

Under conditions of acute or chronic hypoxia, or during LOAD or when exposed to exogenous Aβs, APP levels and both β–S and γ–S activities raise significantly thereby increasing Aβs production and release (Perez et al., 2010; Dal Prà et al., 2011, 2014a,b; Takami and Funamoto, 2012). This might be due to Aβ_42_-os entering the nuclei and binding Aβ-interacting domains (AβIDs) in the *APP* and β–*S* genes promoters sequences causing their transcriptional activation (Bailey et al., 2011; Maloney and Lahiri, 2011; Barucker et al., 2014). Lastly, Aβ-activated microglia release cytokines, like IL-1β or IFN-γ + TNF-α, that induce cultured adult human astrocytes to synthesize and secrete Aβ_40_ and Aβ_42_ (Blasko et al., 2000).

## Aβs as receptorial ligands

As mentioned, an exposure to fibrillar or soluble Aβ_25−35_ elicits an excess production, accumulation, and secretion of Aβ_42_ and Aβ_42_-os in cultured NAHAs (Armato et al., 2013a; Dal Prà et al., 2015a). The primary molecular mechanism(s) underlying this exogenous Aβs ⇒ endogenous Aβs self-induction in NAHAs was (were) initially totally and still partly is (are) unclear. It appeared that exogenous Aβs interacted with “something” located at the outer surface of the cells' plasma membrane (Kam et al., 2014; Jarosz-Griffiths et al., 2016). At the same time, the question arose whether this Aβs self-inducing feed-forward mechanism worked in *human* neurons too, as it had been shown to do in Aβ-exposed rat cortical neurons and mouse hippocampal slices (Marsden et al., 2011). So would Aβs bind and activate the signaling of one or more receptors? So far, several receptors have been indicated to interact with Aβs (Table 1). Nevertheless, since Aβs are the unique ligands for none of them, these Aβ•receptor interactions have been debated. Yet, once bound to Aβs some of the receptors did undergo internalization and accumulated intracellularly (Kam et al., 2014; Jarosz-Griffiths et al., 2016). For example, highly specific soluble Aβ-os•CaSR complexes were shown to gather together and form patches at the plasma membrane of NAHAs prior to be internalized (Dal Prà et al., 2014a,b, 2015b). Conversely, most of the fibrillar Aβ•CaSR complexes could not be internalized because of intrinsic mechanical hindrances and their persistent signaling likely altered crucial cellular functions with noxious and/or lethal consequences. This happened also in engineered SK-N-BE neuroblastoma cells over expressing the whole p75^NTR^ receptor which bound fibrillar Aβs (Perini et al., 2002). Indeed, p75^NTR^ is also over expressed in the hippocampi of full-blown LOAD patients (Chakravarthy et al., 2012).

**Table 1:** **Receptor interactions with various Aβ forms**.

**Aβ forms**	**Receptor**	**References**
Aβ_42_ monomers	Insulin-like growth factor-1 receptor (IGF-1R)	Giuffrida et al., 2012
Aβ_42_ monomers	Low-density lipoprotein receptor-related protein 1 (LRP1)	Shibata, 2000; Kanekiyo et al., 2013, 2012
Aβ_42_ monomers	Low-density lipoprotein receptor (LDLR)	Castellano et al., 2012
Aβ_42_ monomers	Macrophage receptor with collagenous structure (MARCO)	Brandenburg et al., 2010
Aβ_42_ and Aβ_40_ monomers	Advanced glycation end products receptor (RAGE)	Du et al., 2012
Aβ_42_ and Aβ_40_ monomers	Apolipoprotein E (ApoE) receptor	Liu et al., 2013
Aβ_42_ monomers, Aβ_42_-os	α7 nicotinic acetylcholine receptor (α7nAChR)	Wang et al., 2000; Jurgensen and Ferreira, 2010
Aβ_40_ monomers, Aβ_42_-os	Cellular prion protein (PrP^*C*^)	Nygaard and Strittmatter, 2009; Pflanzner et al., 2012
Aβ_42_	Formyl peptide receptor (FPR1) Formyl peptide receptor-like 1 (FPRL1)	Iribarren et al., 2005; Doens and Fernandez, 2014
Aβ globulomers	P/Q-type Ca^2+^ channels	Nimmrich et al., 2008
Aβ_42_-os, Aβ_40_-os	Frizzled (Fzd) receptor	Magdesian et al., 2008
Aβ_42_-os	Insulin receptor	Zhao et al., 2008
Aβ_42_-os	α-amino-3-hydroxy-5-methyl-4- isoxazole propionic acid receptor (AMPAR)	Zhao et al., 2010
Aβ_42_-os	Amylin 3 (AMY3) receptor	Fu et al., 2012
Aβ_42_-os	NMDA-type glutamate receptor	Shankar et al., 2007
Aβ_42_-os, Aβ fibrils	Calcium-sensing receptor (CaSR)	Ye et al., 1997; Conley et al., 2009; Dal Prà et al., 2014a,b, 2015b
Aβ_42_-os, Aβ fibrils	p75 neurotrophin (p75^NTR^) receptor	Perini et al., 2002; Chakravarthy et al., 2012
Aβ fibrils	SCARA1/2 (microglia) receptor	Wilkinson and El Khoury, 2012
Aβ fibrils	SCARB2/CD36 receptor	Stewart et al., 2010
Aβ fibrils	Toll-like receptor 2 (TLR2)	Doens and Fernandez, 2014
Aβ fibrils	Complement receptor type 3 (CR3)	Doens and Fernandez, 2014

A clue on the topic was offered by observations that a mixture of three cytokines (i.e., TNF-α, IL-1β, and IFN-γ) or soluble Aβ_40_ or fibrillar Aβ_25−35_ or Aβ_1−42_ induced a MEK/ERK1/2-mediated surplus NO production in NAHAs that could be fully suppressed when the cells were co-treated with a CaSR antagonist (or *calcilytic*) like NPS 89686 or NPS 2143 (Nemeth, 2002; Chiarini et al., 2005; Dal Prà et al., 2005; Armato et al., 2013a). Such results prompted us to investigate CaSR's interactions with Aβs in human cortical astrocytes and neurons (Armato et al., 2012, 2013a).

## The CaSR in the several neural cell types

The readers looking for more details about the features of the CaSR are referred to other contributions in this special issue. Briefly, the CaSR is a member of family C of G-protein-coupled receptors (GPCRs). Its huge (~612 amino acids) extracellular N-terminal domain, named Venus Flytrap (VFT), is linked via a cysteine-rich region to seven transmembrane α-helices (TM1–TM7) joined together by extracellular and intracellular loops altogether forming the 7TM region. Two domains of the CASR's intracellular C-terminal tail are necessary for its expression at the cell surface and its composite signaling functions which are mediated by G-proteins (Armato et al., 2012). The two huge VFT lobes of functional CaSR homodimers bind orthosteric (type I) *agonists* like Ca^2+^ (the physiological ligand), various other divalent or trivalent cations, polyamines, and aminoglycoside antibiotics (Silve et al., 2005; Armato et al., 2012; Zhang et al., 2015). The allosteric (type II) CaSR ligands, like aromatic L-α-amino acids and highly selective *agonists* (or *calcimimetics*) and *antagonists* (or *calcilytics*) bind various 7TM sites (Nemeth, 2002; see also below). The CaSR swiftly senses any change in the [Ca^2+^]_e_ (Nemeth, 2002). Orthosteric type I agonists switch CaSR's signaling on owing to a rearrangement of its 7TM region permitting the receptor's C-tails to interact with various G proteins. The manifold CaSR's signaling pathways involve (i) second messenger-producing enzymes (e.g., adenylyl cyclase); (ii) phospholipases A2, C, and D; (iii) protein kinases (e.g., AKT, PKCs, MAPKs,); (iv) Ca^2+^ influxes via TRPC6-encoded receptor-operated channels; and (v) transcription factors (TFs; reviewed in Zhang et al., 2015). Like other GPCRs, CaSRs display the “*ligand-biased signaling*” feature, i.e., a specific CaSR signaling pathway may be stably preferred over the others according to the ligand involved (Leach et al., 2015). Here we will briefly consider some pathophysiological effects of CaSR's signaling regarding the CNS.

The CaSR is expressed by all types of neural cells and by the endothelial cell and pericytes of the cerebral vessels with a variable intensity, which for example is greater in the hippocampus (Chattopadhyay et al., 2000, 2008; Yano et al., 2004; Noh et al., 2015). In addition, cultured NAHAs also express functional CaSRs, less intensely when proliferating but more strongly when in mitotic quiescence. In any case, CaSR expression is unaffected by changes in the growth medium Ca^2+^ levels (Dal Prà et al., 2005).

**Figure 2:**
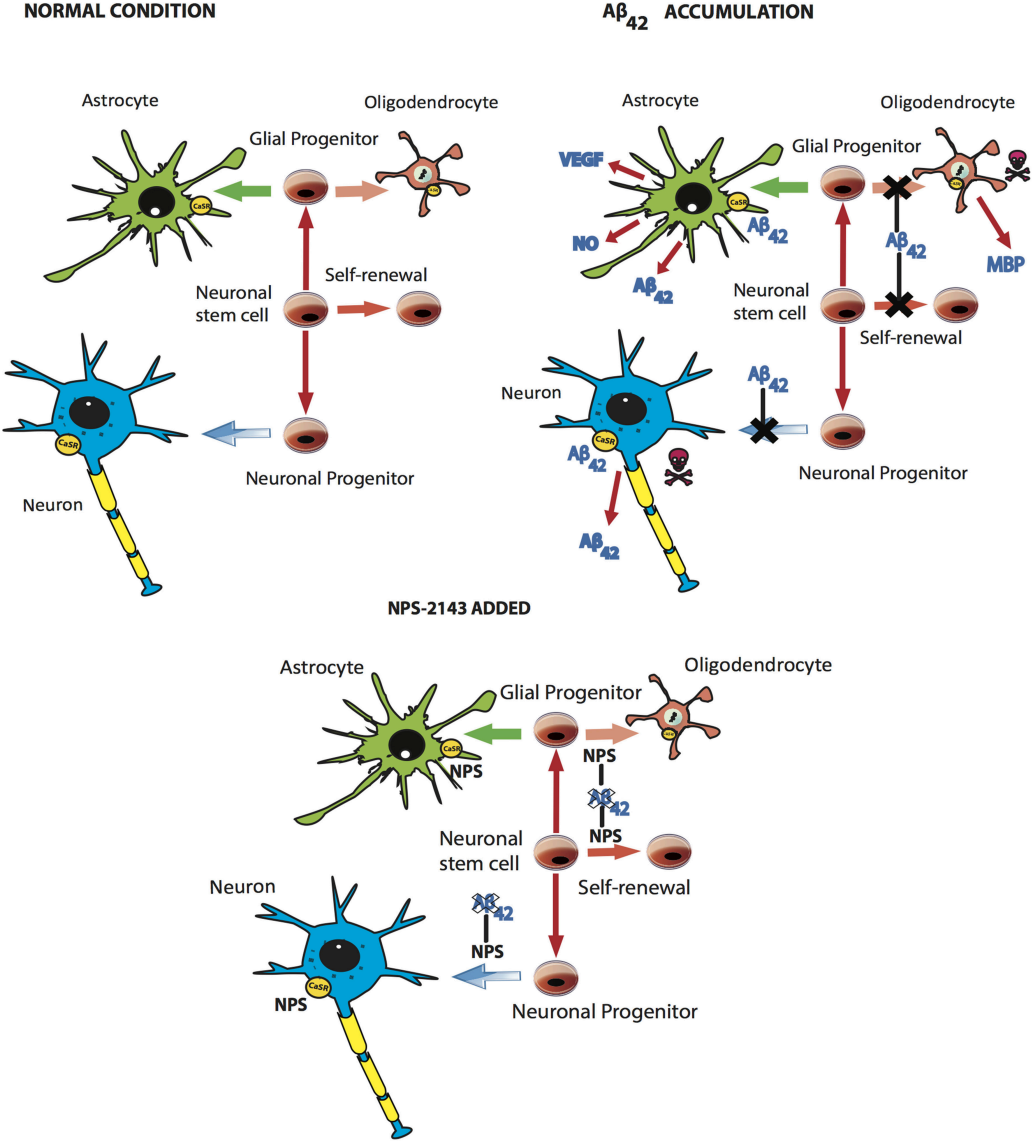
**The main neural cell types origins, and the effects of Aβ_42_-os accumulation without or with an added calcilytic. Top left:** During development and in the adult, neurogenesis starts from NSCs that self-renew while giving birth to neurons and glial progenitors. From the latter (also named NG2 cells) stem both astrocytes and oligodendrocytes. All these cell types express the CaSR (see the text for details). **Top right:** When Aβ_42_-os start accumulating in the brain tissues they soon block the NSCs self-renewal and differentiation of both neurons and oligodendrocytes from their respective precursors. The interactions of Aβ_42_-os with the CaSRs (*yellow ovals*) elicits a surplus production/release of Aβ_42_-os from neurons and astrocytes, of NO and VEGF-A from astrocytes, and of MBP and Aβ_42_-os (not shown) from oligodendrocytes. All these toxic compounds together with later appearing p-Tau-os (not shown), microglial proinflammatory cytokines, and hypoxia/ischemia due to damaged micro vessels eventually cause an increasing death of neurons and oligodendrocytes (*flanking skull and crossbones*). **Bottom center:** The addition of calcilytic NPS 2143 (short termed here as *NPS*) thwarts all of the toxic effects elicited by Aβ_42_-os•CaSR signaling like surplus secretion and diffusion of additional Aβ_42_-os, NO, and VEGF-A, hindered differentiation of NSCs, and most of all, the death of neurons and oligodendrocytes, vascular damage, the later p-Tau-os appearance, and likely microglial activation (the latter two not shown). The findings on neurons and astrocytes indicate the feasibility of calcilytics as anti-LOAD therapeutics capable of halting Aβ_42_-os self-promoting and self-maintaining mechanisms (Dal Prà et al., 2015a).

CASR is a key player in genetic regulation of Ca^2+^ homeostatic system (Kapur et al., 2010). In addition, CaSR performs relevant roles outside the Ca^2+^ homeostatic system, as for example in the CNS (Riccardi and Kemp, 2012). Besides upholding local ionic homeostasis, brain cells' CaSRs modulate the proliferation, differentiation, and migration of neurons and oligodendrocytes during development; axonal and dendritic growth; axons myelination; neurons' and glial membrane excitability; olfactory and gustatory signal integration; presynaptic external Ca^2+^ signaling at *neocortex* nerve terminals; synaptic plasticity; and neurotransmission during perinatal and adult life. Importantly, an altered expression and/or dysfunction of the CaSR, as observed in CNS diseases like AD and ischemia/hypoxia/stroke, also deeply affects CaSR-dependent neurophysiological processes (Figure 2) (Chattopadhyay et al., 1999; Vizard et al., 2008; Bandyopadhyay et al., 2010; Chen et al., 2010; Armato et al., 2012, 2013a; Ruat and Traiffort, 2013; Kim et al., 2014; Dal Prà et al., 2014a,b, 2015a; Bai et al., 2015; Noh et al., 2015; Tharmalingam et al., 2016).

The first clue about a potential role for the CaSR in AD pathophysiology was the degeneration of hippocampal neurons ensuing Aβ-induced peaks of cytosolic (intracellular) Ca^2+^ concentration ([Ca^2+^]_i_) (Brorson et al., 1995). A second clue was the assumed ability of fibrillar Aβ_25−35_ or Aβ_1−40_ to open Ca^2+^-permeable non-selective cation channels (NSCCs) in hippocampal neurons of wild type (WT) CaSR^+∕+^ rats but not of CaSR^−∕−^ rats (Ye et al., 1997). The authors posited that Aβs could bind the CaSR since like polyamines they are endowed with orderly spaced arrays of positive charges. However, the same authors had previously observed hefty changes in pipette cations concentrations in cell-attached recordings and their replacement with Ca^2+^ had not affected channel amplitude or reversal potential (Ye et al., 1996a,b). Taken together, these results would have suggested the channel was alike permeable to K^+^ and Na^+^ or, alternatively, impermeable to cations like a Cl^−^ channel. However, the authors did not test his hypothesis. In this regard, several other authors have reported NSCCs being activated by *decreases* of calcium or other CaSR agonists (Hablitz et al., 1986; Xiong et al., 1997; Immke and McCleskey, 2001; Smith et al., 2004; Lu et al., 2010; Ma et al., 2012). Overall such findings do not corroborate the suggestion that Aβs activate NSCCs in neurons. Rather, Aβs would lessen the likelihood of NSCC openings or possibly activate Cl^−^ channels like probably the data from Ye et al. (1997) had demonstrated.

Afterwards, Conley et al. (2009) investigated the association of *CASR* gene variations in AD susceptibility using a cohort of 692 AD cases and 435 controls. A polymorphic dinucleotide repeat marker within intron 4 associated with AD, while three non-synonymous SNPs within exon 7 of the *CASR* gene associated with AD only in non-APOE ε4 carriers. In addition, TF activation assays revealed that both apoE ε4 and ε3 (but not ε2) and exogenous Aβ_1−42_ bound and activated CaSR's signaling. The authors concluded that the CASR plays a role in AD susceptibility in the absence of the APOE ε4 allele(s).

Subsequently, the formation of Aβs•CaSR complexes and their endocytosis was shown to occur in NAHAs by using the highly specific *in situ* proximity ligation assay (Dal Prà et al., 2014a,b, 2015b). As aforesaid, such Aβs•CaSR complexes elicited a surplus production and secretion of Aβ_42_ and Aβ_42_-os from cortical NAHAs and HCN-1A neurons (Armato et al., 2013a). These observations imply that all types of human neural and cerebrovascular cells are susceptible to the neurotoxic effect(s) elicited by Aβ•CaSR signaling.

*CASR* gene transcription is regulated by its promoters P1 and P2 which bind several TFs. Recently, the role of TFs in a number of genes associated with AD has been studied in detail. Interestingly, the *CASR* gene promoters bind several TFs which are involved also in the expression of AD-related genes. Thus, there exists a deeper than previously thought connection of CaSR expression regulation with AD pathophysiology (see Table 2 and references in it). Although CaSR mRNA and protein levels have not yet been investigated in human AD brains, it is likely that *CASR's* expression be altered in AD because of its co-regulation by some of the TFs implicated in the disease.

**Table 2:** **Comparison between ***CaSR*** and AD-related genes transcriptional regulators**.

**Gene**	**Transcription factor**	**References**
*CaSR*	SP1/3^*^, **AP1**, **STAT1**/**3, NF**κ**B, TFIID**, Vitamin D, GCM-2	Santpere et al., 2006; Hendy et al., 2013
*APP*	**SP1**, **AP1**, **STAT1**/**3, NF**κ**B**, USF, CTCF, HSF1, SP1-like, UBP, **HIF-1**α**, CREB**, GATA1	Theuns and Van Broeckhoven, 2000; Santpere et al., 2006; Chen et al., 2013
BACE1	**SP1, STAT1/3, NF**κ**B**, HIF-1α, PPAR γ	Santpere et al., 2006; Wen et al., 2008; Chen et al., 2013
*PSEN1*	**SP1**, Ets, **CREB**	Theuns and Van Broeckhoven, 2000; Santpere et al., 2006; Chen et al., 2013
*APOE*	**SP1**, **TFIID**, **AP-2**, URE3BP, PPARγ	Theuns and Van Broeckhoven, 2000; Santpere et al., 2006; Chen et al., 2013;
*MAPT*	**SP1, AP-2**, Nrf1, MTF1, MBF1, MepI, GCF	Santpere et al., 2006; Caillet-Boudin et al., 2015

* *Shared transcription factors are in bold characters*.

Besides the CaSR, Aβ_42_-os simultaneously link to many other surface receptors (Table 1) activating their signaling systems and changing ion balances prior to be endocytosed by all types of CNS cells. In so doing, Aβ_42_-os spark a dense clutter of cellular responses including mitochondrial over release of toxic ROS, Ca^2+^ surges via NMDARs' activation driving further mitochondrial releases of ROS, and production of toxic p-Tau oligomers (p-Tau-os) (Mao and Reddy, 2011; Müller et al., 2011; Swerdlow, 2011; Kam et al., 2014; Jarosz-Griffiths et al., 2016). The outcomes are the disconnection of neuronal networks—a cause of cognitive deterioration—and the damage and death of susceptible neurons eventually leading to full blown AD (Crimins et al., 2013; Kayed and Lasagna-Reeves, 2013; Medeiros et al., 2013).

However, the earliest asymptomatic stages of AD are still hard to detect because the build-up of highly toxic, synapse destroying Aβ_42_-os inside and outside neurons and astrocytes is imperceptible until senile plaques and NFTs remain undetectable (West et al., 2004; Selkoe, 2008a,b; Ferreira and Klein, 2011; Klein, 2013; Medeiros et al., 2013; Dal Prà et al., 2015a). Thus, in the course of several years the neurotoxic Aβ_42_-os spread stealthily from LEC's layer II to higher cognitive cortical areas (Khan et al., 2014) and the emergence of AD's typical hallmarks (Figure 1A). As it will be discussed below, these events are related to Aβ_42_-os•CaSR interactions whose signaling mechanisms are likely to underlie the developing amyloidosis in AD brains and hence have crucial therapeutic implications.

Some authors surmise a prion-like mechanism fostering the Aβ_42_-os (and p-Tau-os) diffusion in AD brains (Nussbaum et al., 2013; Morales et al., 2015). Aβ_42_-os extracted from AD brains could be passed on from retinoic acid–differentiated human SH-SY5Y donor neurons to similarly differentiated SH-SY5Y recipient neurons (Nath et al., 2012; Hallbeck et al., 2013). Most important, an Aβ_42_-os propagation within the brains of Tg APP-model mice or WT rats or marmoset (*Callithrix jacchus*) monkeys also obtained via injections of AD brain extracts or punctures made with steel wires coated with the same extracts (Maclean et al., 2000; Meyer-Luehmann et al., 2006; Eisele et al., 2009; Langer et al., 2011; Hamaguchi et al., 2012; Rosen et al., 2012). In such animal models, the diffusion of the injected Aβ_42_-os followed the same route as developing AD pursues in humans, i.e., LEC layer II ⇒ perforant pathway ⇒ hippocampal dentate gyrus and CA3 area ⇒ upper cortical regions (Morrison and Hof, 2007; Khan et al., 2014). Besides, a cerebrovascular amyloidosis was induced after a delay of various months by intraperitoneal injections of 1000-fold higher doses of Aβ_42_-os-charged mouse brain extracts (Eisele et al., 2010). The mechanisms by which misfolded Aβ_42_-os (and p-Tau-os) propagate within the brain are undetermined (Moreno-Gonzalez and Soto, 2011). So far, the prion-like intrabrain spreading potential of Aβ_42_-os appears to be feeble as compared to the infectious capabilities proper of true prions and to require a direct contact with the neural cells (Aguzzi and Rajendran, 2009; Irwin et al., 2013). In addition, an Aβ_42_-os amplifying mechanism has simultaneously to operate in order to assist the prion-like diffusion of the brain amyloidosis (Brettschneider et al., 2015). Hypothetically, this amplifying mechanism might result from or be aided by the Aβ_42_-os own self-induction and self-spreading properties due to their interaction with the CaSRs of neurons, astrocytes, and other brain cell types (Dal Prà et al., 2015a).

## Aβ•CaSR signaling promotes intra- and extracellular toxic Aβ_42_/Aβ_42_-os overloads

By using as preclinical models *in vitro* cortical nontumorigenic NAHAs and postnatal HCN-1A neurons brought to a complete proliferative quiescence, exogenous Aβ_25−35_-os and Aβ_1−42_-os were shown to bind the plasma membrane-inserted CaSRs with a high specificity. These bonds activated CaSR's intracellular signaling pathways which in turn elicited a whole set of pathophysiological effects in both cell types, including the death of the HCN-1A neurons (summarized in Table 3; (Dal Prà et al., 2011, 2014a,b, 2015a,b; Armato et al., 2013a; Ruat and Traiffort, 2013). As mentioned, an Aβ-os•CaSR-activated MEK/ERK-dependent pathway mediated NO overproduction in NAHAs; the same signaling stabilized the HIF-1α•HIF-1β TF which then entered the astrocytes' nuclei to trigger a VEGF-A surplus production and secretion (Dal Prà et al., 2005, 2014b).

**Table 3:** **Harmful effects Aβ•CaSR signaling elicits in human neurons and astrocytes**.

**Cell type**	**Stimulus**	**Pathological effect**	**Effect of adding calcilytic to Aβs**	**Effect of calcimimetic alone**
Neurons, astrocytes	Aβ_42_-os Aβ fibrils	Overproduction and diffuse intracellular accumulation of endogenous Aβ_42_ monomers and Aβ_42_-os due to an increased β-S and γ-S activity and (likely) to decreases in Aβ proteolysis	Total suppression of intracellular accumulation of Aβ_42_ monomers and Aβ_42_-os due to increased Aβ-os proteolysis (no effect on increased β-S and γ-S activities)	No apparent intracellular accumulation of Aβs
Neurons, astrocytes	Aβ_42_-os Aβ fibrils	Concurrent Aβ_40_-os intracellular accumulation	Modest decrease of Aβ_40_-os intracellular accumulation	n. d.^*^
Neurons, astrocytes	Aβ_42_-os Aβ fibrils	Surplus secretion of Aβ_42_-os, but not of Aβ_40_-os, along the Golgi/*trans*-Golgi pathway and axons ⇒ extracellular Aβ_42_/Aβ_40_ ratios values shift to the cytotoxic range	Total suppression of surplus release of Aβ_42_-os along the Golgi/*trans*-Golgi pathway and axons, but increased release of Aβ_40_-os⇒ extracellular Aβ_42_/Aβ_40_ ratios values remain in the normal range (NPS 2143 by itself exerts no effect on basal Aβ_42_-os secretion)	Significant surplus secretion of Aβ_42_-os
Neurons	Aβ_42_-os Aβ fibrils	Slow yet progressive death by apoptosis of the human cortical neurons (*in vivo* this is the cause of cognitive decline; Nelson et al., 2012).	Neurons remain alive and kicking	n. d.
Astrocytes	Aβ_42_-os Aβ fibrils	NAHAs survive and keep making and releasing neuron-harming compounds (see below)	No apparent effect on survival	n. d.
Astrocytes	Aβ fibrils	Increased activity of the glycogen synthase kinase (GSK)-3β, one of the main Tau kinases (Armato et al., 2013b).	Total suppression of the surge of GSK-3β activity (Armato et al., 2013b).	n. d.
Astrocytes	Aβ_42_-os Aβ fibrils	Stabilization and nuclear translocation of the HIF-1α•HIF-1β transcription factor ⇒ expression of VEGF-A, APP, and BACE1 genes ⇒ heightened synthesis/secretion of VEGF-A and Aβ_42_/Aβ_42_-os	HIF-1α destabilization ⇒ deep yet transient decrease of nuclear HIF-1α•HIF-1β transfer ⇒ no surplus production/release of VEGF-A, APP, and Aβ_42_/Aβ_42_-os	n. d.
Astrocytes	Aβ_42_-os, Aβ fibrils	Significant yet transient surge of total CASR protein	Downregulation of total CaSR protein: modest and transient with NPS 2143 alone but fast, intense and persistent with Aβs + NPS 2143	No change in total CaSR protein
Astrocytes	Aβ_42_-os Aβ fibrils	Induction and MEK/ERK-dependent activation of GTP cyclohydrolase-1 (GCH1) ⇒ production of BH4 (tetrahydrobiopterin) ⇒ dimerization and activation of the concomitantly induced NO synthase (NOS)-2 ⇒ excess release of NO	Inactivation of GCH1 ⇒ lack of BH4 ⇒ no dimerization and activation of the concomitantly induced NO synthase (NOS)-2 ⇒ no overproduction of NO	n. d.

* *n.d., not determined*.

By contrast, the mechanisms of the increased synthesis, accumulation, and release of Aβ_42_-os elicited through Aβ-os•CaSR signaling in both NAHAs and HCN-1A neurons are not as yet fully understood and currently under investigation. In regard to this topic, an upregulation of the CaSR and an intensified Aβ-os•CaSR signaling induced the death of neurons in rodent models of cerebral ischemia/hypoxia/stroke (Kim et al., 2014). This is a second instance besides LOAD in which Aβ-os•CaSR signaling kills neurons.

## Neurotoxic Aβ•CaSR interactions in other glial and cerebrovascular cells

The extremely complex mammalian CNS harbors several distinct cell types. Generally, LOAD discussions focus most on neurons, less on microglia, but leave the other cell types in the shade. However, several authors have tried to broaden this restricted viewing by putting astrocytes into the fray (Busciglio et al., 1993; Blasko et al., 2000; Chiarini et al., 2005; Li et al., 2011; Zhao et al., 2011; Armato et al., 2013a; Dal Prà et al., 2015a). Indeed, in our work we have been using both NAHAs and HCN-1A neurons as separate models to clarify the responses evoked by an exposure to exogenous Aβ-os. Hence, we do not deem astrocytes being more important players than neurons in LOAD promotion. Instead, we have been endorsing a holistic view, i.e., all CNS cell types are likewise important players both in CNS physiology and LOAD pathophysiology. Consequently, we review below relevant knowledge concerning the CaSR with respect to LOAD in the remaining glial cell types and cerebrovascular cells.

### Oligodendrocytes

Oligodendrocytes precursors (NG-2 glial cells) generate also protoplasmic astrocytes (Figure 2) and maybe neurons, and receive synaptic inputs. These precursors become functionally impaired and/or damaged with aging (reviewed in Cai and Xiao, 2016). When NSCs' differentiation is aimed at the oligodendrocyte lineage, the expression and activity of NSCs' CaSRs are up-regulated, which favors their expansion and differentiation (Chattopadhyay et al., 2008). Human oligodendrocytes keep amplifying their numbers up to 5 years of age, when they amount to ~5–10% of the total glia. Thereafter, their turnover remains negligible. Typically, oligodendrocytes' myelin production and myelin sheaths' upkeep quickly adapt to ongoing needs, e.g., learning activities—a feature promoting neural plasticity. During AD development, at neurotoxic levels (i.e., in the μM range) Aβ-os switch off the Wnt signaling of the precursors thereby hindering their differentiation into oligodendrocytes (Figure 2; Barateiro et al., 2016). In advanced AD, soluble and fibrillar Aβs and/or p-Taues/NFTs together with ongoing oxidative stress and neuroinflammation cause oligodendrocytes' to dysfunction and die via apoptosis (reviewed in Cai and Xiao, 2016). Consequently, myelin sheaths break down first in the hippocampus and fornix, and later in the other involved areas (Roth et al., 2005). The breakdown of myelin sheaths releases myelin basic protein (MBP), which by itself triggers a neurotoxic discharge of NO from cortical NAHAs. This MBP effect is synergistically amplified by a mixture of three proinflammatory cytokines (TNF-α, IL-1β, and IFN-γ) or by soluble Aβ_40_ (Chiarini et al., 2005). So far, the specific neurotoxic effects of Aβ-os•CaSR signaling have not been investigated in human oligodendrocytes, although doing it would be worthwhile. Interestingly, mature oligodendrocytes are able to express APP and to produce and secrete both Aβ_42_ and Aβ_40_ (Skaper et al., 2009). Therefore, besides neurons and astrocytes, oligodendrocytes are the third potentially relevant source of endogenous Aβ_42_-os in LOAD—a source hitherto disregarded perhaps because of its progressive cytotoxic damage and destruction. Thus, a crucial role of Aβ-os•CaSR signaling in the neurotoxic responses and demise of oligodendrocytes in AD remains to be proven.

### Microglia

At variance with neural cells, microglia arise from circulating myeloid monocytes which migrated into the CNS during gestation to act there as macrophage equivalents. In their physiologically “quiescent” phenotype, microglia promote brain development and relevantly upkeep neural environment homeostasis, immunological surveillance, modulation of neuronal proliferation and differentiation, pruning of synapses, and clipping of apoptosing neurons (Saijo and Glass, 2011; Harry, 2013). Microglia become “activated” in response to neural tissue injury and then start sweeping up debris from degenerated neurons and infectious agents (when present), hence favoring tissue repair (Yang et al., 2010; Lee et al., 2011; Derecki et al., 2013; McGeer and McGeer, 2015). Moreover, in AD microglia are persistently activated, keep engulfing extracellularly accumulating fibrillar and soluble Aβs, and surround and infiltrate dense core senile plaques where they promote Aβs fibrillation (reviewed in Rosen et al., 2012). Additionally, both Aβ fibrils and Aβ_42_-os trigger the microglia to secrete proinflammatory cytokines, (e.g., IL-1β, TNF-α, IFN-γ), chemokines (Lindberg et al., 2005; Färber and Kettenmann, 2006; Kawanokuchi et al., 2006; Saijo and Glass, 2011; Heneka et al., 2013; Prokop et al., 2013), ROS, NO, and even N-terminally truncated Aβ-os (Nagele et al., 2004; Mawuenyega et al., 2010; Oberstein et al., 2015). The interaction of ROS and NO generates hyper toxic peroxynitrites (ONOO^−^). Therefore, both microglia and astrocytes contribute to kindle and keep going the chronic neuroinflammation proper of LOAD brains. Furthermore, the microglial cytokines bind and activate their specific receptors on the surface of the astrocytes which are thus stimulated to produce and release additional amounts of Aβ-os, NO, and VEGF-A besides the amounts of the same agents generated by the astrocytes own Aβ-os•CaSR signaling (Dal Prà et al., 2005, 2015a; Chiarini et al., 2010). In turn, these microglia-elicited astrocytes' secretions sustain and/or intensify microglia activation, starting vicious cycles of astrocytes ⇔ microglia reciprocal interactions. They also stimulate the adjoining ANTs to produce additional Aβ_42_-os that keep spreading and via Aβ-os•CaSR signaling elicit the release of (a) further amounts of Aβ_42_-os from neurons and astrocytes; (b) NO and VEGF-A from astrocytes; (c) MBP and Aβ_42_-os from oligodendrocytes; and (d) proinflammatory cytokines, chemokines, ROS, NO, and N-terminally truncated Aβs from microglia (Blasko et al., 2000; Lindberg et al., 2005; Kawanokuchi et al., 2006; Mandrekar-Colucci and Landreth, 2010; Zhao et al., 2011; Prokop et al., 2013; Oberstein et al., 2015). Thus, self-maintaining and spreading vicious cycles of reciprocal interactions between microglia and ANTs' members keep exerting significant toxic effects on all neural cell types (Nath et al., 2012) strongly promoting as a result LOAD progression (Rojo et al., 2008; Nordberg, 2014). However, likely for cytotoxic reasons microglia's inflammogenic role decreases with the progressing of AD (Mizuno, 2012), and it may even become less relevant than astrocytes' role due to the greater numbers, stronger resistance to toxic agents, and longer-lasting functional activation of the astrocytes (reviewed in Rosen et al., 2012).

Moreover, the specific outcomes of the interactions between exogenous Aβ_42_-os and microglial CaSRs of WT and AD-model rodents are still undefined notwithstanding their potential relevance to LOAD therapy (McGeer and McGeer, 2015). Notably, rat microglia express a functional CaSR capable of modulating a Ca^2+^-activated K^+^ channel (Chattopadhyay et al., 1999; Yano et al., 2004). In this regard, we recall that the BV-2 immortalized murine microglial cell line was reported to constitutively produce and release Aβs. Moreover, and remarkably, adding exogenous Aβ_25−35_ or lipopolysaccharide increased the production and secretion of Aβs from BV-2 microglial cells (Bitting et al., 1996). The authors did not assess the CaSR's role in this process. Nevertheless, the findings of Bitting et al. (1996) and Oberstein et al. (2015) indicate microglia as a likely fourth *source* of Aβ-os in LOAD brains. As far as we know, no study about the CaSR in *human* microglia has been reported. Therefore, the role of Aβ-os•CaSR signaling in microglia deserves further investigations.

### Cerebral vessels

Astrocytic processes' end-feet envelop the cerebral micro vessels forming functional NVUs which govern the delivery of nutrients and oxygen required for the activities of ANTs' neurons (Figure 1B). In LOAD, accumulating Aβ42-os, Aβ fibrils, and NO harm the cells of the cerebral vessels eventually causing the onset of a cerebral amyloid angiopathy (CAA) which helps advance LOAD (Nelson et al., 2016). CAA's degenerative changes include perivascular ring-like Aβs casts staving off astrocytic processes' end-feet, increased vessel walls stiffness, weakened responses to astrocyte-released vasodilator agents, impeded neoangiogenesis, and changed BBB permeability, altogether causing local hypoxic/ischemic lesions (Kimbrough et al., 2015; Love and Miners, 2015). The latter favor the production/release of Aβ42-os surpluses, likely via Aβ42-os•CaSR signaling from adjoining ANTs' components thus hastening cognitive decline (reviewed in Helman and Murphy, 2016). The endothelial cells of human aorta and other vessels express the CaSR (Ziegelstein et al., 2006). By inference, the endothelia and other cellular components of the human brain vessels' walls should also express the CaSR. Then again, to our knowledge, no study has specifically addressed the potential toxic effects of Aβ-os•CaSR signaling on the cerebrovascular walls pericytes, smooth muscle cells, and endothelial cells in LOAD. Finally, yet importantly, LOAD-damaged blood vessels can block neurogenesis from NSCs in the subventricular zone and hippocampus (Figure 2) thereby thwarting the processing and storage of new memories (Licht and Keshet, 2015).

In summary, the interactive dysfunctional responses of all brain-resident cell types evoked via Aβ-os•CaSR signaling are likely to play significant roles in the promotion of LOAD (Brorson et al., 1995; Dal Prà et al., 2015a).

## Aβ-os•CaSR interactions advance LOAD progression

**Figure 3:**
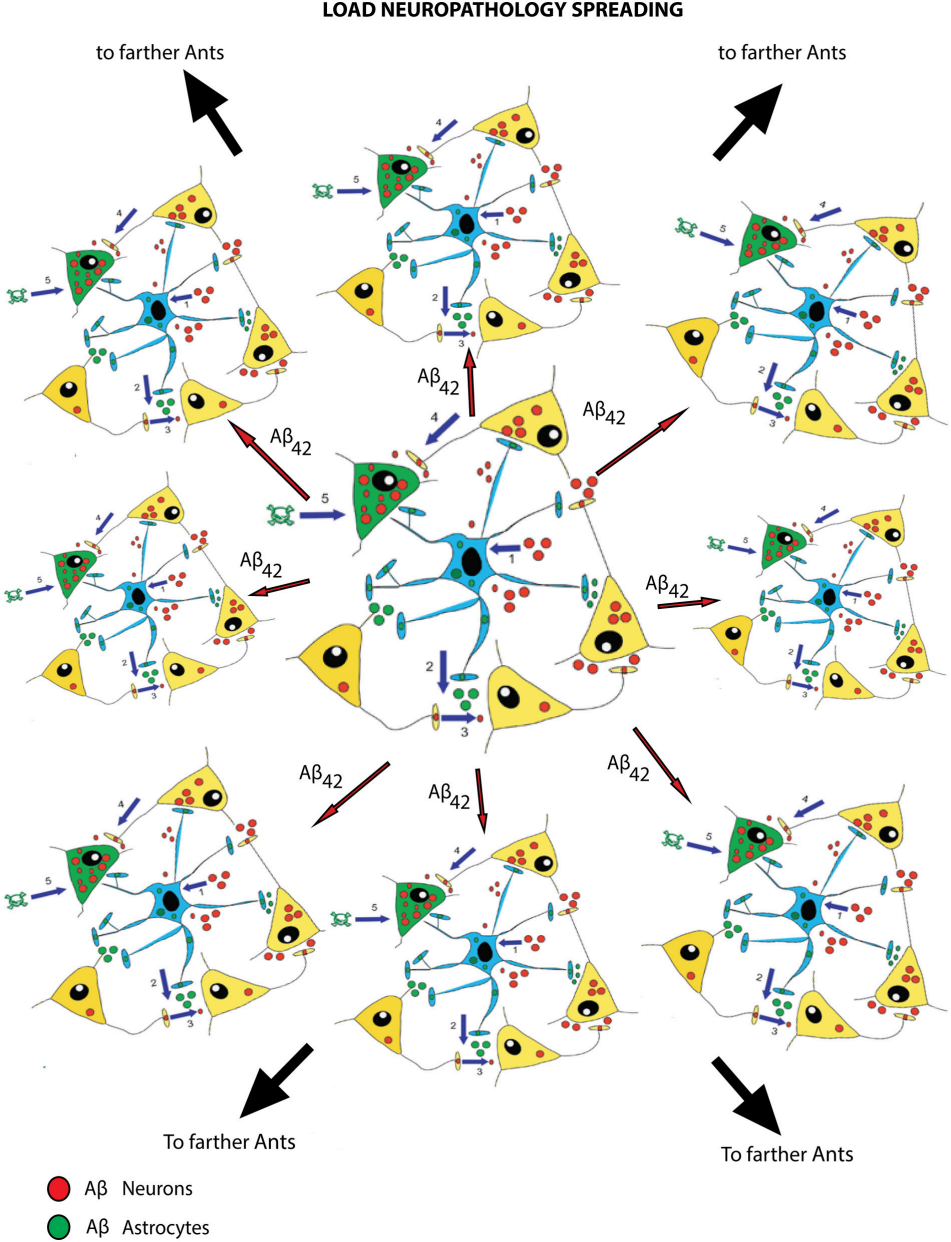
**Propagation of LOAD neuropathology to neighboring astrocyte-neurons teams (ANTs)**. The cartoon shows that an excess of exogenous Aβ_42_-os (here short-termed as Aβ_42_) supposedly reaches first the team of neurons and astrocytes (ANT) at the center and binds their CaSRs (not detailed) triggering signals that end up increasing the secretion of newly produced endogenous Aβ_42_-os (*red and green circles*) from all of the ANT's cellular members (*# 1–5*). Blue arrows indicate the diffusion of Aβ_42_-os from neurons to astrocytes (red solid circles) and from astrocytes to neurons (green solid circles). Numbers 1–5 also suggest possible sequences of events both intra- and inter-ANTs. While the involved cells undergo cytotoxic changes, including the early death of some neurons (*in green color with skull and crossbones aside*), the newly released Aβ_42_-os spread and reach both neighboring and remoter ANTs *(short and long red arrows)*, starting via Aβ_42_-os•CaSR signaling new cycles of surplus production and secretion of endogenous Aβ_42_-os. The latter will disperse and engage nearby and still farther away ANTs (not shown) again triggering the same kind of Aβ_42_-os•CaSR signaling-triggered pathological responses, including additional Aβ_42_-os oversecretion and neuronal deaths. Thus, Aβ_42_-os spread can affect local ANTs (as embodied here by the *short and long red arrows*) or remoter ANTs via projecting axons carrying the Aβ_42_-os (as exemplified here by the *big black arrows*).

During AD development, ANTs' vital functions can turn into grievously troublesome ones. When the “client” neurons and their “master” astrocytes of small *foci* in the LEC's layer II overproduce Aβ_42_ monomers, they start releasing Aβ_42_-os into the synaptic spaces surrounded by the astrocytes' shrouding end-feet and into the extracellular milieu. When Aβ_42_-os spread to the CA1 area, the formation of new memories begins to fail (Bushong et al., 2002). We already mentioned that ANTs' neurons and astrocytes are endowed with a variety of Aβ_42_-os-binding receptors, including the CaSR (Table 1). Next, released Aβ_42_-os scatter from the ANTs of origin to contiguous ANTs, in which they bind and activate the local neurons' and astrocytes' CaSRs (Dal Prà et al., 2015a). As a result, both cell types start producing and releasing additional Aβ_42_-os, which will keep spreading and targeting the CaSRs of neurons and astrocytes of remoter ANTs (Figure 3). Remarkably, Aβ_42_-os diffuse not only by contiguity, but also by apparent “jumps” because the blighted hippocampal neurons project their long axons carrying Aβ_42_-os surpluses to the *neocortex* of far-off cerebral lobes (Figure 1A). Thus, reiterating feed-forward cycles of this kind which sustain and amplify themselves via Aβ_42_-os•CaSRs interactions end up recruiting the neurons and astrocytes of ever-increasing numbers of ANTs. The latter will make and release still more Aβ_42_-os which will spread to even farther off ANTs. Thus, from the LEC LOAD neuropathology would reach through this basic molecular mechanism upper cerebral cortical areas (Dal Prà et al., 2015a). Aβ_42_-os•CaSRs and Aβ_42_-os•PRP^C^s interactions would next favor the gradual appearance of p-Tau-os which at some later point will acquire via still undefined mechanism(s) the ability to self-induce themselves and spread independently of Aβ_42_-os. Afterwards, both toxic drivers would hasten AD progression toward its gloomy conclusion (Dal Prà et al., 2015a).

## Pharmacological CaSR modulators and LOAD

Various synthetic phenyl alkylamines derivatives endowed with two-to-four aromatic rings and NH^3+^ groups selectively act either as CaSR's type II allosteric *agonists* (or *calcimimetics*; e.g., NPS R-568, Cinacalcet, and AMG 416) or *antagonists* (or *calcilytics*; e.g., NPS 89636, NPS 2143). Such agents shift to the right or to the left, respectively, the CaSR's response curve to changes in extracellular Ca^2+^ concentration ([Ca^2+^]_e_) (Nemeth, 2002; Saidak et al., 2009; Widler, 2011). These CaSR modulators bind distinct sites in the 7TM region—both calcimimetics and calcilytics between TM6 and TM7, but calcilytics alone between TM3 and TM5 (Petrel et al., 2004). The full therapeutic potential of CaSR modulators has yet to be gauged in human ailments (Saidak et al., 2009; Widler, 2011; Ward et al., 2012; Nemeth, 2015). These agents too can promote the “*ligand-biased signaling*” according to the specific cell type considered—a feature that might favor target-specific therapeutic approaches (Davey et al., 2012; Leach et al., 2015)

### Calcimimetics

NPS R-568 and Cinacalcet are presently the best paradigms of allosteric CaSR agonists as they hinder PTH secretion (Nemeth, 2004, 2013). In clinical settings, Cinacalcet has been and still is used to manage primary hyperparathyroidism and secondary hyperparathyroidism due to chronic kidney disease (CKD). It has been used particularly in patients in chronic dialysis, although in some of these cases it failed to be effective (Nemeth and Goodman, 2015; Brunaud et al., 2016). Cinacalcet also averts or reverses parathyroid hyperplasia in rats and functionally rescues CaSR's loss-of function mutations (Nemeth, 2004, 2013; Miller et al., 2012; Nemeth and Shoback, 2013; Palmer et al., 2013; Nemeth and Goodman, 2015; Mayr et al., 2016). However, since CaSR expression is ubiquitous, one should not overlook that calcimimetics (and calcilytics) may exert PTH-independent effects in tissues, brain included, other than the parathyroid glands (Massy et al., 2014). As an example, a Cinacalcet-triggered protracted CaSR signaling curtailed the mitotic activity and interfered with the remodeling and barrier function of oesophageal epithelial cells via catenin-cadherin complexes disruption, actin cytoskeletal changes, and CaSR reallocation to the nuclei (Abdulnour-Nakhoul et al., 2015). Various pieces of evidence discussed in previous sections have denoted the CaSR's involvement in AD onset and progression. Remarkably, calcimimetic NPS R-568 mimics at least one pathological effect of Aβs•CaSR signaling: it significantly increases the amount of Aβ_42_-os secreted by cortical NAHAs (Armato et al., 2013a; Dal Prà et al., 2015a; Table 2). The potential clinical implications of this NPS R-568 effect deserve further assessment.

### Calcilytics

Compounds like NPS 2143, NPS 89636, Calhex, etc., desensitize the CaSR to [Ca^2+^]_e_ changes and characteristically increase PTH secretion (Nemeth, 2004, 2013). Various calcilytics were initially tested as therapeutics for postmenopausal osteoporosis. But, they lacked effectiveness because they elicited a PTH oversecretion which stimulated in parallel both osteogenesis and osteolysis. This stopped any further clinical testing concerning a potential anti-osteoporosis activity of calcilytics (Nemeth, 2004, 2013; Nemeth and Shoback, 2013; Nemeth and Goodman, 2015). Novel indications for calcilytics are (i) idiopathic hypercalciuria; and (ii) autosomal dominant hypocalcaemia due to CaSR's gain-of-function mutations; as for the latter condition calcilytic NPS P-795 is being tested as a therapeutic in clinical trials (White et al., 2009; Letz et al., 2010; Park et al., 2013; Nemeth, 2015; Nemeth and Goodman, 2015). In addition, calcilytics may mitigate the airways hyper responsiveness and inflammation proper of asthma (Yarova et al., 2015). Calcilytics also inhibit the cellular hyper proliferation typical of pulmonary artery idiopathic hypertension (Yamamura et al., 2012, 2015)

A further potential indication of calcilytics is LOAD (Armato et al., 2013a). In fact, we showed that in cultured *human* untransformed cortical NAHAs and HCN-1A neurons calcilytics NPS 2143 and NPS 89696 counteracted *all* the noxious consequences—death of the neurons included—brought about by Aβs•CaSR signaling (Table 3; Armato et al., 2013a; Dal Prà et al., 2014a,b, 2015a). These preclinical findings indicate that, by hindering the Aβs•CaSR signaling at the level of neurons, of all glial cell types, and of cerebrovascular cells, calcilytics would effectively suppress (or at least significantly mitigate) the intracerebral propagation of the amyloidosis and its concurrent neurotoxic effects. By keeping the neurons alive and functioning, calcilytics would safeguard the patients' cognitive faculties. Most remarkably, calcilytics would be the so far unique anti-LOAD therapeutics simultaneously targeting a number of LOAD-promoting processes which Aβs•CaSR signaling triggers in all types of CNS cells (Armato et al., 2012, 2013a; Dal Prà et al., 2014a,b, 2015a). Calcilytics reduce neuronal death also in animal models of ischemia/hypoxia/stroke, i.e., in conditions that increase Aβ-os production in the affected brain area(s) (Kim et al., 2014; Bai et al., 2015). These findings too strongly substantiate our hypothesis about the crucial role of the CaSR in AD.

Here, some pharmacological notations are in order. Being lipid-soluble, calcilytics cross the BBB, can be administered by any route (oral, etc.), and in the presence of exogenous soluble Aβ-os or fibrillar Aβs (which also release Aβ-os) selectively antagonize CaSR's signaling and intensely down regulate the CaSRs of human cortical astrocytes, and likely neurons and other CNS cells (Armato et al., 2013a; Dal Prà et al., 2015a). Calcilytic NPS 2143 is well tolerated by rodents (Nemeth, 2002; Kim et al., 2014). Recent NPS 2143 derivatives, which stimulate PTH secretion less intensely, were well withstood by human subjects during phase I and phase II clinical trials aimed at assessing the drugs' anti-osteoporosis activity (in such instances no consideration was given to brain-related effects; Nemeth and Shoback, 2013; John et al., 2014). Of late, the NMDA receptor inhibitors Memantine and Nitromemantine and the Fyn kinase inhibitor Saracatinib (AZD0530) were suggested as therapeutics to offset the neurotoxic actions brought about by extracellularly gathering Aβ-os (Talantova et al., 2013; Kaufman et al., 2015). It should be realized that the calcilytics' target, i.e., the CaSR, holds an *upstream* place with respect to NMDARs and Fyn. Therefore, calcilytics' ability to hinder any extracellular Aβ_42_-os build-up would as well prevent any downstream Aβ_42_-os harmful effects involving NMDARs and Fyn. Moreover, by keeping the extracellular Aβ_42_/Aβ_40_ ratio values within the physiological range, calcilytics would thwart any cytotoxic effects and hindrance of NSCs differentiation (Figure 2) and of functions necessary for neurogenesis to occur in the dentate gyrus subgranular zone. Calcilytics would also safeguard the structural and functional integrity of cognition-critical upper cerebral cortical areas (Choi et al., 2013; Lee et al., 2013; Barateiro et al., 2016). In short, calcilytics would preserve the patients' ability to store and retrieve memories and to cope with daily needs, thus improving her/his life's quality and prospects.

Calcilytics' failure as therapeutics for osteoporosis due to the double-edged effects of PTH was a stroke of ill-luck (Nemeth, 2004, 2013; Nemeth and Shoback, 2013; Nemeth and Goodman, 2015). In addition, calcilytics' potential side effects—e.g., mild hyperparathyroidism in humans, hypertension in rats—shied people away from considering their use in clinical settings. However, calcilytics' rather mild side effects must be carefully weighed against the harsh fact that *symptomatic LOAD inexorably kills the patient cognitively several years before her/his actual physical demise*. Hence, just as anticancer chemotherapeutics are used notwithstanding their potential side effects, once clinical trials have proven calcilytics therapeutic effectiveness, their side effects will be a trivial toll against preventing/stopping LOAD progression.

## Conclusions and future perspectives

Astrocytes' and neurons' pathophysiology in LOAD brains are quite intricate and specific for each animal species, brain area, aging phase, and stage of the illness. Therefore, a deeper understanding of AD-related metabolic events occurring in human cortical untransformed astrocytes and neurons has helped and will help identify ground breaking therapeutic approaches to LOAD. Differently from neurons, human astrocytes survive for lengthy terms the exposure to toxic amounts (in the μM range) of soluble or fibrillar Aβs while undergoing complex and only partially understood functional changes collectively defined as *activation*. The latter include, amongst others, alterations of (a) the Aβ•α7-nAChR signaling affecting the intra- and intercellular Ca^2+^ signaling and gliotransmitters secretion, and (b) the Aβ•CaSR signaling triggering a surplus production and secretion of neurotoxic Aβ_42_-os, VEGF-A, and NO. However, since the CaSR is endowed with panoply of intracellular signaling pathways, we undertake that not all of the toxic metabolic effects prompted by Aβ•CaSR signaling have been yet identified in human neural cells. Moreover, the interactions of Aβs with receptors other than the CaSR and/or Aβs-mediated non-receptorial mechanisms add other neurotoxic factors (e.g., proinflammatory cytokines, chemokines, ROS, etc.) which confound the picture. Collectively, these manifold metabolic responses reveal the deep involvement of astrocytes in LOAD's promotion. This view is strengthened by a recent report demonstrating that, while human neurons release only Aβ_1−42_, human astrocytes secrete a remarkable amount of N-terminally truncated Aβs, including Aβ_3−42_ moieties that are transformed into the utterly toxic pE-Aβ_3−42_ (Gunn et al., 2010; Morawski et al., 2014; Oberstein et al., 2015). However, here one should not overlook that oligodendrocytes and microglia and the cellular components of cerebral vessels also express the CaSR. Therefore, toxic Aβ•CaSR interactions do also occur at the level of the latter cell types which may induce the production and release of further amounts of Aβ_42_-os thereby helping advance AD progression. This field is worth exploring further because white matter damage, neuroinflammation, and local hypoxia/ischemia/stroke play relevant roles in LOAD pathophysiology.

It is noteworthy that allosteric CaSR antagonists or calcilytics can suppress upstream all the downstream toxic consequences of Aβ•CaSR signaling in both human neurons and astrocytes, and likely might do the same in all other neural and vascular cell types of the CNS. These findings make us posit that calcilytics would effectively prevent LOAD amyloidosis from spreading. Moreover, by hindering Aβ_42_-os accumulation and diffusion calcilytics would prevent also the ensuing appearance and spread of the p-Tau-os and their lethal cooperation with Aβ_42_-os (Dal Prà et al., 2005, 2014a,b, 2015a; Armato et al., 2013a).

In conclusion, these findings attest the need to increase our understanding of CaSR's pathophysiology in all types of human untransformed neural cells. In fact, one cannot disregard that meanwhile LOAD is flaring up worldwide in an epidemic-like fashion. Therefore, it would be timely to validate the anti-LOAD effectiveness of calcilytics in clinical trials recruiting aMCI and/or early symptomatic patients.

## Author contributions

AC, ID, and UA contributed equally to the manuscript's conception. DL made searches and helped with the manuscript.

## Funding

This work was supported by the Italian MIUR (Ministero dell'Istruzione, dell'Università e della Ricerca) via FUR 2013, FUR 2014, FUR 2015, and FUR 2016 funds allotted to AC, ID, and UA.

### Conflict of interest statement

The authors declare that the research was conducted in the absence of any commercial or financial relationships that could be construed as a potential conflict of interest.

## Abbreviations

α7-nAChR(s): α7-nicotinic acetylcholine receptor(s)
AD: Alzheimer's disease
(a)MCI: (amnestic) minor cognitive impairment
ANT(s): astrocyte-neuron team(s)
APOE: apolipoprotein E
APP: Aβ precursor protein
Aβ(s): amyloid-β peptide(s)
Aβ_42_-os: amyloid-β_42_ oligomers
β-S: BACE1/βS, β-secretase
CAA: cerebral amyloid angiopathy
CaSR: calcium-sensing receptor
CKD: chronic kidney disease
EOFAD: early onset familial (autosomal dominant) AD
fMRI: functional Magnetic Resonance Imaging
GABA: γ-amino butyric acid
γ-S: γ-secretase
GFAP: glial fibrillary acidic protein
GPCRs: G-protein-coupled receptors
LDL: low density lipoprotein
LEC: lateral entorhinal *allocortex*
LOAD: late-onset (sporadic) AD
LRP1: LDL receptor-related protein 1
LTP: long-term potentiation
MBP: myelin basic protein
NAHAs: normofunctioning adult human astrocytes (from cerebral cortex)
n.d.: not determined
NFTs: neurofibrillary tangles
NMDA: N-methyl-D-aspartate
NO: nitric oxide
NSCs: neural stem cells
NVU(s): neurovascular unit(s)
p75^NTR^: p75 neurotrophin (receptor)
PAR-4: prostate apoptosis response-4
pE: pyroglutamate
PET: positron emission tomography
PKA: protein kinase A
PSEN1/2: presenilin 1/2
(p)-Tau-os: hyperphosphorylated Tau protein oligomers
(p)-Tau(es): hyperphosphorylated Tau protein(s)
PTH: parathyroid hormone
TF(s): transcription factor(s)
Tg: transgenic
VEGF-A: vascular endothelial growth factor A
VFT: Venus Fly Trap
WT: wild-type
7TM: seven transmembrane α-helices region.
